# Vertical transmissibility of small ruminant lentivirus

**DOI:** 10.1371/journal.pone.0239916

**Published:** 2020-11-18

**Authors:** Juscilânia Furtado Araújo, Alice Andrioli, Raymundo Rizaldo Pinheiro, Lucia Helena Sider, Ana Lídia Madeira de Sousa, Dalva Alana Aragão de Azevedo, Renato Mesquita Peixoto, Ana Milena Cesar Lima, Edgar Marques Damasceno, Samara Cristina Rocha Souza, Maria Fátima da Silva Teixeira

**Affiliations:** 1 Northeast Network in Biotechnology, State University of Ceará, Fortaleza, Ceará, Brazil; 2 Embrapa Goats and Sheep, Sobral, Ceará, Brazil; 3 Veterinary Sciences, State University of Ceará, Fortaleza, Ceará, Brazil; 4 Scholarship for Regional Scientific Development of the National Council for Scientific and Technological Development (DCR-CNPq/FUNCAP), level C, Brasilia, Distrito Federal–DF, Brazil; 5 Animal Science, Federal University of Piauí, Teresina, Piauí, Brazil; 6 Zootechnics, State University of Acaraú Valley, Sobral, Ceará, Brazil; 7 Biological Sciences, State University of Acaraú Valley, Sobral, Ceará, Brazil; 8 State University of Ceará, Fortaleza, Ceará, Brazil; Universidade Federal de Minas Gerais, BRAZIL

## Abstract

This study aimed to evaluate by means of *Nested* Polymerase Chain Reaction (*n*PCR), co-cultivation and sequencing, with genetic comparison between strains (mother/newborn), the occurrence of vertical transmission of Small Ruminant Lentiviruses (SRLV) from naturally occurring nannies infected for their offspring. For the detection of SRLV seropositive progenitors, blood was collected from 42 nannies in the final third of gestation in tubes with and without anticoagulant. The diagnostic tests used were Western Blot (WB) and *n*PCR. During the period of birth, the same blood collection procedure was performed on 73 newborns at zero hours of birth, with the same diagnostic tests. Seventeen blood samples from seven-day-old kids, proven positive for SRLV by *n*PCR, chosen at random, were subjected to coculture in goat synovial membrane (GSM) cells for 105 days. The pro-viral DNA extracted from the cell supernatant from the coculture was subjected to *n*PCR. For DNA sequencing from the *n*PCR products, nine positive samples were chosen at random, four nannies with their respective offspring, also positive. Each sample was performed in triplicate, thus generating 27 *n*PCR products of which only 19 were suitable for analysis. Among the 42 pregnant goats, in 50% (21/42) pro-viral DNA was detected by *n*PCR, while in the WB, only 7.14% (3/42) presented antibodies against SRLV. Regarding neonates, of the 73 kids, 34 (46.57%) were positive for the virus, using the *n*PCR technique, while in the serological test (WB), three positive animals (4.10%) were observed. The coculture of the 17 samples with a positive result in the *n*PCR was confirmed in viral isolation by amplification of the SRLV pro-viral DNA. When aligned, the pro-viral DNA sequences (nannies and their respective offspring) presented homology in relation to the standard strain CAEV Co. It was concluded that the transmission of SRLV through intrauterine route was potentially the source of infection in the newborn goats.

## Introduction

Small ruminant lentiviruses (SRLV) are viral agents that affect sheep and goats, and that share homologous sequences in the organization of the genome [1]. These viral agents belong to the *Retroviridae* family, which are able to transcribe ribonucleic acid (RNA) to deoxyribonucleic acid (DNA), through the action of the reverse transcriptase enzyme, and often cross the interspecific barrier between sheep and goats, with great genetic variability [2,3].

Like the human immunodeficiency virus (HIV), SRLV have regions of polymorphism that help them evade the barriers of the immune system [4]. For presenting homologous sequences, between the V4 region and the HIV V3 region [5], and also at the level of surface glycoproteins with it, with a similar function in modulating conformational changes, SRLV infections are considered useful models in studies for HIV and other retroviruses [1].

Structural (*gag*, *pol* and *env*) and accessories genes (*vpr*, *rev* and *vif*) make up the genome of SRLV [6]. Among these, the *gag* gene is one of the most conserved regions of the viral genome [7], and worldwide it is the target of molecular analysis [3,6,8–12], allowing to properly characterize the circulating strains in the infected animal's organism due to its ideal minimum genetic variability, compared to other conserved regions of the genome [13,14].

Although the main entry point for SRLV into the animal's body is via the lactogenic route [15,16], and through contact between healthy and infected animals [17], viral compartmentalization, with migration of the virus from the bloodstream to other organs, like the uterus, becomes an important risk factor for the disease [7–18].

In addition, the hypothesis has been raised that intrauterine transmission is likely to occur, since there are reports of the detection of pro-viral DNA in tissues of the uterus, oviduct and ovary, demonstrating tropism of the SRLV in these regions [19]. Associated with this, there is also the fact that umbilical cord cells from small ruminants have been shown to be permissible to infection *in vitro* by these viral agents [20]. Thus, it is possible to deduce that a similar situation may occur *in vivo*, since uterine goat epithelial cells have already shown susceptibility to infection by SRLV in *in vivo* tests [21]. This hypothesis is reinforced with the detection of pro-viral DNA in six offspring from parents carrying SRLV [22].

In this context, the objective of this work was to evaluate, by means of *Nested* Polymerase Chain Reaction (*n*PCR), reinforced by co-culture, the occurrence of vertical transmission of SRLV from naturally infected mothers to their offspring. In addition, the objective was to use genetic sequencing to prove that the isolated strains would be SRLV.

## Material and methods

The study was carried out in the dairy herd of Embrapa Goats and Sheep, in the city of Sobral located in the northern region of the state of Ceará, Brazil. This research was approved by the Animal Use Ethics Commission (CEUA) of Embrapa Goats and Sheep (protocol #010/2018), following the guidelines of the National Council for Animal Experimentation Control (CONCEA, Law 11794 of October 8, 2008) and other subsequent regulatory resolutions.

It is noteworthy that the herd used for this study has been submitted to a SRLV control program, with the following actions being applied: the semi-annual serological diagnosis of all animals by Western Blot (WB), according to the methodology described in the literature [23]; separation of the mother's goats soon after birth, without the mother having any contact with them [24] and the supply of colostrum submitted to heating at 56° C for one hour [25].

### Blood collection and diagnostic test

Initially, for the detection of SRLV seropositive progenitors, blood was collected from 42 nannies in the final third of gestation through the venipuncture of the jugular, using a vacuum system, with 5mL tubes with and without anticoagulant (Ethylenediamine tetraacetic acid—EDTA). After collection, the tubes without anticoagulant were centrifuged in a centrifuge at room temperature at 1500g for 10 minutes to separate the blood serum, subsequently subjected to the Western Blot test [23]. The anticoagulant tubes were destined for the extraction of deoxyribonucleic acid (DNA) according to standard methodology [26] and submitted to *Nested* PCR (*n*PCR) [27]. At birth, before ingesting colostrum, without any contact between the mother and the young, 73 kids were subjected to the same blood collection procedure, with subsequent performance of the same diagnostic tests.

All deliveries were assisted with the immediate removal of the offspring close to the mother in order to avoid any contact, and consequently contamination. Blood collection took place less than an hour after birth.

### Coculture with goat synovial membrane (GSM)

Secondary cultures of goat synovial membrane (GSM) cells were obtained by explant from goat kidney proven to be negative for SRLV, followed by subcultures by trypsinization of the cells [26]. Seventeen samples of whole blood from seven-day-old kids, proven positive for SRLV by *n*PCR, were collected. In order to avoid contamination and biasing the results, it was chosen, at random, 17 samples to be co-cultivated.

The samples were centrifuged at 1,800g, twice with 1,000 μL of 0.84% ammonium chloride and washed three times with 1,000 μL of PBS 1X (sodium-phosphate buffer: 8g NaCl; 0.2g KCl; 0.2g of KH_2_PO_4_; 1.15g of Na_2_HPO_4_ in 1,000 mL of H_2_O; reagents: Sigma-Aldrich®, USA) to obtain cells from the phagocytic mononuclear system [28]. Then, 1,000 μL of minimal essential medium (MEM—Gibco®, USA) was added to these isolated cells, added with 1% amphotericin B (Sigma-Aldrich®, USA), 2% penicillin and streptomycin (P/S—Gibco®, USA) and 10% fetal bovine serum (FBS—Gibco®, USA). Subsequently, the samples were distributed in tissue culture plates of 24 wells in the quantity of 100 μL, in four repetitions in the concentration of 2.0 x 10^5^ cells/μL in each well. The wells were added with 1,900 μL of minimum essential medium (MEM) treated under the same conditions. For follow-up during the culture period, eight control wells were established: four being the negative control (C-) composed only of GSM cells, and four representing the positive control (C +) with GSM cells infected with a standard strain of SRLV (CAEV Co), with an initial titre of 10^−4,8^ TCID_50_/mL. The plates were stored in a 5% CO_2_ environment, at 37°C, for four days, with a change of macrophage culture medium in 48 hours.

After that period, GSM cells were added, in the 9th passage, at a concentration of 2.0 X 10^5^ cells/μL, kept in an incubator under the same conditions. Periodic changes of medium were performed every seven days and cellular trypsinization every 21 days, totaling 105 days of culture. Throughout the culture period (105 days), nine collections of supernatant containing non-adherent cells were performed, separated by treatment, to perform the *nested* polymerase chain reaction (*n*PCR) in order to detect the presence of pro-viral DNA. After 63 days of the start of cultivation, the plates were duplicated, generating replicas, and after 17 days of duplication, only the replicas were stained with violet crystal (0.1%) to visualize cytopathic effects, such as syncytium, cell destruction and crenated cells.

### Pro-viral DNA extraction from cell supernatant and *n*PCR

The extraction of pro-viral DNA from cell supernatant from co-culture was performed based on proteinase K and ethanol (Sigma-Aldrich®, USA), according to the methodology already described in the literature [26]. A first round of *n*PCR was performed, followed by a second round in order to amplify a final fragment of 185 base pairs (bp) of pro-viral DNA, which corresponds to the SRLV *gag* gene. However, from the samples destined for sequencing (five newborns and their respective mothers) a first round was carried out, and from this product, three second rounds were carried out on different days, thus obtaining replicates of all samples. All oligonucleotide primers were produced based on the standard CAEV-Co sequence (M33677.1) [29] (Table 1).

**Table 1:** Sequences of primers used in the *Nested* Polymerase Chain Reaction (*n*PCR) with the size of the amplified fragments.

*Gag* Gene	*Primers*	Sequences 5’ → 3’	Fragments (pb)
1^st^ *round*	*Gag* 1	CAAGCAGCAGGAGGGAGAAGCTG	297
	*Gag* 2	TCCTACCCCCATAATTTGATCCAC	
2^nd^ *round*	*Gag* 3	GTTCCAGCAACTGCAAACAGTAGCAATG	185
	*Gag* 4	ACCTTTCTGCTTCTTCATTTAATTTCCC	

In addition to the tested samples and for each round of amplification, a negative control (without DNA) and a positive control referring to CAEV Co (standard viral sample kindly provided by the Federal Rural University of Pernambuco, from the Laboratoire Associé de Recherches sur les Petits) Ruminants—INRA—ENVL—France).

The *n*PCR reactions were performed in a thermocycler (BIO-RAD, T100TM Thermal Cycler) in a total volume of 50 μL, containing buffer (10 mM tris-HCl, 50 mM KCl and 1.5 mM MgCl_2_—Sigma-Aldrich®, USA), 100 μM of each dNTP (Sigma-Aldrich®, USA), 20 pmol of each primer, 2U of *Taq* Platinum DNA polymerase (Thermo Fisher®, USA); 3μL of sample in the first round and 1μL of first round product in the second round.

Amplification by *n*PCR occurred at 94° C for five minutes, 35 cycles of 94° C for one minute, 56° C for one minute and 72° C for 45 seconds, followed by a final extension at 72° C for seven minutes. The amplified samples and the controls (positive and negative), were submitted to electrophoresis in 2% agarose gel (Sigma-Aldrich®, USA), stained with ethidium bromide (Sigma-Aldrich®, USA) and visualized in ultraviolet transilluminator (UVP, Benchtop UV Transiluminator M-26) [27].

### Sequencing and analysis

For the DNA sequencing of the *n*PCR products, nine positive samples were chosen, five of them with their respective mothers, also infected for SRLV (nanny A, B, C and D and offspring 1, 2, 3, 4, 5). In the selection of the samples to be sequenced, the kids were chosen randomly, with subsequent selection of the respective mothers, with the purpose of not trending the results. Thus, the nine samples and their respective replicates, totaling 27 *n*PCR products were sequenced. The sequencing was performed using the Sanger method, via the Applied Biosystems® 3500 Genetic Analyzer platform. To obtain consensus sequences, editing and alignment, the BioEdit Sequence Alignment Editor® programs [30] and MEGA software version 7.0 [31] were used.

To obtain the consensus sequences, the forward and reverse strings of each sample and their respective replicates were aligned, followed by the removal of the primers. Then, the consensus sequences generated from each sample and the viable replicates were analyzed (each sample was worked in triplicate, except for two samples in which it was not possible to obtain three complete sequences) and aligned to come to a single sequence (denominated the average consensus). Subsequently, the average consensus sequences of the mothers were aligned with those of their respective offspring, and both (offspring and mothers) with the sequences of the standard strains CAEV Co and MVV K1514, available on GenBank with the respective access numbers M33677.1 and M10608.1. Also included in the alignment were some Brazilian BR CNPC sequences (Genbank accession number EU300976, EU300977, EU300978, EU300979).

## Results

Among the 42 pregnant goats, in 50% (21/42) pro-viral DNA was detected by *n*PCR. However, serologically, through the WB, only 7.14% (3/42) presented antibodies against SRLV. In relation to neonates, 46.57% (34/73) of these animals were positive for the virus, using the *n*PCR technique, whereas via WB only 4.10% (3/73) were seropositive.

When co-culturing samples from 17 neonates with a positive result in the *n*PCR, their positivity was confirmed via isolation by amplifying the SRLV pro-viral DNA. It should be noted that after 21 days (1^st^ collection), 64.7% (11/17) of the samples showed a positive result in the *n*PCR (Table 2).

**Table 2:** Result of the *Nested* Polymerase Chain Reaction (*n*PCR) of the pro-viral DNA extracted from the supernatant collected over 105 days of goat synovial membrane (GSM) cell culture and cells from the phagocytic mononuclear system of blood samples from neonates.

ANIMALS	COLLECTION								
	1^st^	2^nd^	3^rd^	4^th^	5^th^	6^th^	7^th^	8^th^	9^th^
C-	-	-	-	-	-	-	-	-	-
C+	+	+	+	+	+	+	+	+	+
1	-	-	-	-	+	+	+	-	-
2	+	-	-	+	+	-	-	+	+
3	+	-	-	-	+	+	+	-	-
4	+	-	+	-	+	+	-	-	+
5	+	-	-	+	-	+	+	+	+
6	+	-	-	-	-	+	-	-	+
7	-	-	-	+	-	+	+	-	+
8	+	-	-	-	-	+	+	+	+
9	+	-	-	-	-	+	+	+	-
10	+	+	-	-	-	+	+	-	+
11	+	+	-	-	+	+	+	+	-
12	+	-	-	-	-	+	-	+	+
13	-	-	-	+	+	+	+	-	+
14	-	-	-	+	-	-	-	+	+
15	-	-	-	-	+	+	+	+	+
16	+	-	-	+	-	+	+	+	-
17	-	-	-	+	-	+	+	+	+

C-: Negative control with goat synovial membrane cells (GSM); C +; Positive control with CAEV Co strain; (-) negative sample in *n*PCR; (+); positive sample in the *n*PCR.

**Fig 1:**
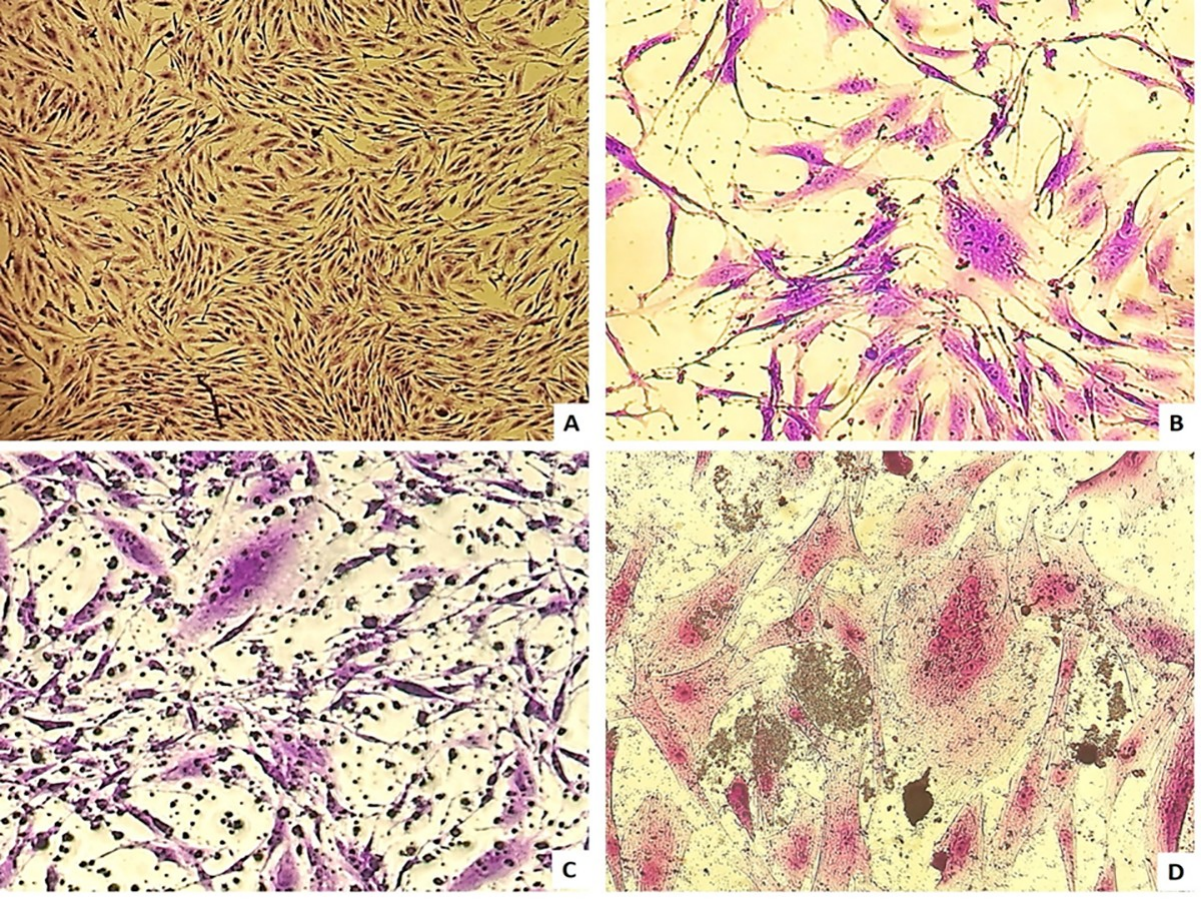
**Coculture with goat synovial membrane cells (GSM) and cells of the mononuclear phagocytic system of blood samples from neonates.** A: Negative control of cell coculture with GSM cells only (100x magnification). B: Coculture of cells from animal 06 with the presence of syncytium (circle) (160x magnification); C: Coculture of cells from animal 09 with visible cell destruction (160x magnification); D: Positive control of cell coculture with GSM cells infected with CAEV Co (200x magnification).

Meanwhile, in the stained replicated cell culture plates, the presence of cytopathic effects (Fig 1) caused by lentiviruses was observed. In all samples, except 4, the presence of a cytopathic effect was observed with levels ranging from very light to very intense (Table 3), thus confirming the occurrence of the virus in the cells of the culture.

**Table 3:** Levels of cytopathic effects characteristic of small ruminant lentivirus (SRLV) in goat synovial membrane (GSM) coculture cells after co-cultivation with cells from the mononuclear phagocytic system of neonates' blood samples.

ANIMALS	CYTOPATHIC EFFECTS		
	Syncytium	Celular destruction	Crenated cells
C^-^	-	-	-
C^+^	+++	+++	+++
1	+	++	+
2	+	+	+
3	+	+	-
4	-	-	-
5	-	++	+
6	+	++	+
7	-	+	+
8	-	+	-
9	-	++	+
10	+	+	-
11	-	+	-
12	-	+	-
13	-	++	+
14	+	+	-
15	-	+	+
16	-	+	-
17	-	+++++	-

C -: Negative control of goat synovial membrane cells (GSM); C +: CAEV Co positive control. (-): No effect; (+): Very light; (++): Light; (+++): Moderate; (++++): Intense; (+++++): Very intense.

When aligning the pro-viral DNA sequences of the nine samples together with their replicates, they were all homologous. Unfortunately, it was not possible to obtain the sequences of some replicates, probably due to the degradation of the DNA samples. Thus, two replicates of the nanny A and of the offspring 1, and one replica of the nanny C and of the kids 2, 3 and 5 did not obtain viable sequencing. The *n*PCR tests were performed on different days in order to exclude any type of contamination.

**Fig 2:**
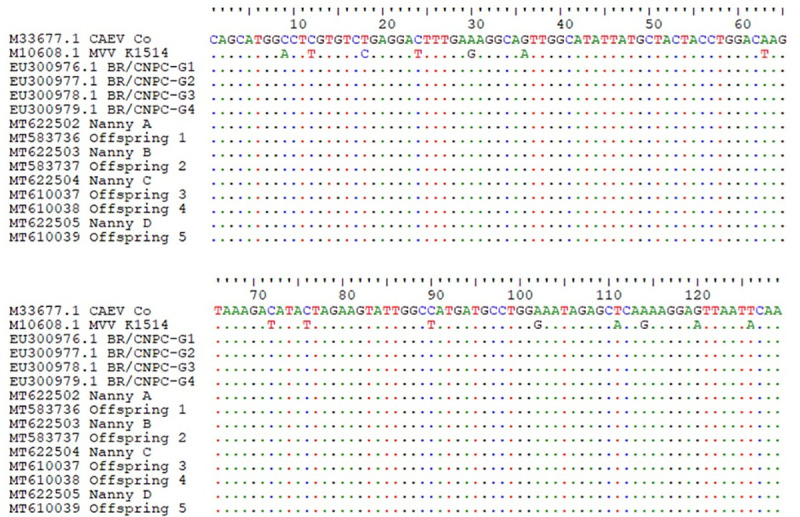
Alignment of the about 129 base pair (bp) fragments of the *gag* gene of the average consensus pro-viral sequences of Small Ruminant Lentivirus (SRLV) from neonates and their respective mothers with standard CAEV Co, MVV K1514 and Brazilian strains described in Genbank.

Subsequently, when defining the consensus sequences for each of the nine samples, and aligning the offspring with its respective mother, it became evident that the strains in question were homologues. Was observed homology when aligning neonate and progenitor consensus sequences with the standard strains CAEV Co and, to a lesser extent, with the MVV K1514. In addition, the strings also showed a homology with the Brazilian strains BR CNPC (Genbank accession number EU300976, EU300977, EU300978, EU300979) (Fig 2).

All new sequences were entered in the GenBank database and are available under check number MT 622502, MT 622503, MT 622504, MT 622505 (nanny) and MT 583736, MT 583737, MT 610037, MT 610038 and MT 610039 (offspring).

## Discussion

The maternal-filial transmission of SRLV over time has shown strong evidence of its occurrence with tropism to the organs of the reproductive system of goats and umbilical cord cells to this group of retroviruses [19,20]. In the present study, results from *n*PCR and Western Blot demonstrated the occurrence of SRLV-positive neonates from mothers infected by these viral agents. These results were confirmed via cell culture, and observation of syncytia and cell destruction, which are some of the main cytopathic effects characteristic of SRLV evidenced in the literature [20,26,32,33].

However, the occurrence of different results between different diagnostic tests is likely to occur in studies with SRLV, being one of the main obstacles in research aimed at elucidating the propagation pathways of these viruses. However, when this situation occurs, in general, it is attributed to the intermittent immune response, which presents variable levels of antibodies in animals known to be infected and which can occur over weeks, months and even years [34].

Thus, molecular tests, such as *n*PCR, are relevant in the identification of infected animals, which in general end up not being detected in serological tests. This fact may be related to late seroconversion or the initial stage of infection, as in these situations this technique is more sensitive [35]. The absence of seroconversion or late seroconversion of an animal infected with SRLV is related to the pathogenicity of the disease, which involves the restriction of replication and viral latency in the form of pro-viral DNA integrated into the monocyte cell genome or in its progenitor cells in the bone marrow [36]. In addition, some genetic components of the hosts can control viral concentration influencing the effectiveness of the immune system [37].

SRLV are retroviruses capable of being detected via molecular techniques in their free form using RT-PCR, or associated with the host cell (pro-viral DNA) [38] and detected by *n*PCR—the technique adopted in the present study. Thus, the occurrence of different results in the *n*PCR of samples from co-culture may be due to the fact that, at times, in the collected supernatant, there may be no cell suspension, with the cells adhering to the plate. If there are no dead cells in the collected supernatant, naturally, a negative result will occur in the *n*PCR, as there is no cell suspension to extract and detect any pro-viral DNA, consequently, there is no consistent detection of positive samples. On the other hand, this also means that, at certain times, more suspended cells occur, thus causing greater detection of pro-viral DNA, as observed in the sixth collection. The greater or lesser number of suspended cells can be explained by the viral load of the sample [39]. As well as the absence of cytopathic effects in the sample 4.

The diagnosis of SRLV is complex, considering that the sensitivity and specificity of each test also differ depending on the samples used. Thus, like other studies, this research confirmed the need for association of techniques and samples in order to improve the sensitivity and specificity of the diagnosis [22,40,41].

In a study about the vertical transmission of SRLV [22], this difference in positivity between the molecular and serological tests was also verified. Out of 17 kids evaluated, six were identified as positive in the *n*PCR, while none of them were positive in the serological tests of enzyme immunoassay (ELISA) and in agar gel immunodiffusion (AGID).

As for the newborn animals, the reduced number of seropositive individuals by the WB can be attributed to a possible fetal immunotolerance, that is, the virus may have infected the animals before the development of immunological competence, possibly resulting in the generation of animals that present persistent viral infection, without detectable immune stimulus, since the response of the antibodies produced to the viral protein limits, in this case, the test's effectiveness [42,43]. If infection of the fetus occurs when the immune system is immature, viral proteins are mistakenly recognized as belonging to the individual (self), which makes the animal immunologically tolerant. This problem occurs with other virus, such as the bovine viral diarrhea virus [44].

However, the fact that newborn animals, born from positive SRLV goats, have pro-viral DNA in their blood, suggests that they were infected in the uterine environment, collaborating with the results already described [45], since in the present research the blood sample of the kid was collected in the first minutes after birth, without any contact with the mother, much less with contaminated utensils.

In another study [46] that evaluated the transmission of the Maedi-Visna virus through the colostrum of infected sheep, it was found that one of the offspring, which was separated immediately after birth and, therefore, before the ingestion of colostrum, was seropositive in the commercial enzyme immunoassay (ELISA). Although the placenta of ruminants is syndesmocorial [47], a fact that prevents the passage of antibodies from the mother to the young, it is not known, to date, if, due to this characteristic, it would also prevent the passage of SRLV to the fetus, or whether fetal infection can occur due to the presence of the virus in other reproductive organs, whose isolation of the virus in the female reproductive system is already reported in the literature [19–21].

In the present study, there was homology in the genetic sequences obtained between neonates and their mothers, and a strong relationship with the standard strain CAEV Co as well as with the Brazilian sequences BR CNPC (GenBank accession number EU300976, EU300977, EU300978, EU300979), thus proving that the isolated strains were from SRLV. This relationship is explained by the fact that the studied gene is one of the most conserved regions of the viral genome [7] and has traditionally been used in molecular analyzes [3,6,8–12]. It should be noted that the use of conserved genes is important because it allows elucidating regional regulatory structures and sequences, a fact that helps the detection of genetic inheritance, assembly of traits and similarities, and the process of development and evolution of an organism [48].

Sequencing is a valuable tool for evidencing similarities in strains and has even been used to confirm interspecific transmission of the virus among small ruminants [35–49]. But, although *n*PCR DNA detection occurred in all nine samples and their respective replicates subjected to sequencing, it is believed that the concentration of pro-viral DNA was not adequate for the viable sequencing of all replicates. Small changes in the genetic sequence of the strains studied were also observed, such as insertions and/or base changes, a fact that has already been evidenced in another study [49]. Although these changes may be related to the natural replication process, as point mutations can generally occur due to reverse transcriptase errors [50], in the present study it is believed that they were due to *in vitro* DNA polymerase errors, as the use of a high fidelity enzyme was not adopted.

The detection of pro-viral DNA by itself and antibodies at zero hours, mainly, as well as the presence of cytopathic effects in the co-culture of blood samples collected from offspring after seven days of birth significantly reinforce the hypothesis of vertical transmission.

Intrauterine lentivirus transmission is documented with HIV in humans [51], where samples of DNA extracted from the stillbirth fetal thymus, spleen and peripheral blood mononuclear cells (PBMC) have proven intrauterine transmissibility through this route, by means of detection of HIV-1 pro-viral DNA by PCR. DNA sequences for HIV-1 were identified in the thymus, spleen and PBMC in 6/8, 8/9, 5/9 samples examined, respectively, with positive results being obtained after 16 weeks of gestation. Thus, this research demonstrates early and frequent transmissions of HIV-1 DNA in the womb.

Taken the results as a whole, it was concluded that the transmission of SRLV via intrauterine route was potentially the source of infection in newborn goats, since viral strains were detected in blood samples from kids immediately after parturition, without any contact. between mother and child, and the presence of cytopathic effects when co-cultivated.

## Supporting information

S1 File (RAR)
